# Perspectives on Reprograming Cancer-Associated Dendritic Cells for Anti-Tumor Therapies

**DOI:** 10.3389/fonc.2014.00072

**Published:** 2014-04-07

**Authors:** Fabian Benencia, Maria Muccioli, Mawadda Alnaeeli

**Affiliations:** ^1^Biomedical Engineering Program, Russ College of Engineering and Technology, Ohio University, Athens, OH, USA; ^2^Diabetes Institute, Ohio University, Athens, OH, USA; ^3^Department of Biomedical Sciences, Heritage College of Osteopathic Medicine, Ohio University, Athens, OH, USA; ^4^Molecular and Cell Biology Program, Ohio University, Athens, OH, USA; ^5^Department of Biological Sciences, Ohio University, Athens, OH, USA

**Keywords:** tumor microenvironment, dendritic cells, vaccines, angiogenesis, targeted delivery

## Abstract

In recent years, the relevance of the tumor microenvironment (TME) in the progression of cancer has gained considerable attention. It has been shown that the TME is capable of inactivating various components of the immune system responsible for tumor clearance, thus favoring cancer cell growth and tumor metastasis. In particular, effects of the TME on antigen-presenting cells, such as dendritic cells (DCs) include rendering these cells unable to promote specific immune responses or transform them into suppressive cells capable of inducing regulatory T cells. In addition, under the influence of the TME, DCs can produce growth factors that induce neovascularization, therefore further contributing to tumor development. Interestingly, cancer-associated DCs harbor tumor antigens and thus have the potential to become anti-tumor vaccines *in situ* if properly reactivated. This perspective article provides an overview of the scientific background and experimental basis for reprograming cancer-associated DCs *in situ* to generate anti-tumor immune responses.

## Introduction

Tumors are composed of cancerous cells and non-cancerous cells such as fibroblasts, endothelial cells, and infiltrating leukocytes. Together with non-cellular components (extracellular matrix proteins), this constitutes the tumor microenvironment (TME). The non-cellular components often support the growth and survival of cancer cells. Moreover, cancer cell growth and survival are influenced by the activation state and responses of infiltrating leukocytes. In particular, leukocytes such as macrophages, T cells, myeloid-derived suppressor cells (MDSCs), and dendritic cells (DCs) have all been shown to participate in tumor development in various settings. For instance, on one hand, chronic inflammation, either induced by infection (e.g., *H. pylori*, Hepatitis virus) or irritants (tobacco smoke, asbestos) constitutes an important risk factor for the development of cancer (1–4). On the contrary, tumor-infiltrating leukocytes, such as cytotoxic T cells can mediate an immune response against the tumor by recognizing tumor antigens and attacking tumor cells in a specific manner (5, 6). Indeed, this is the basis of cancer immunotherapies. Thus, immunosuppression is also able to support tumor growth. Furthermore, existing evidence supports that adaptive immune response influences the behavior of human tumors. *In situ* analysis of tumor-infiltrating immune cells may therefore be a valuable prognostic tool in the treatment of colorectal cancer and possibly other malignancies (7).

There are two main ways in which leukocytes can collaborate with tumor development (i.e., pro-tumorigenic processes): suppression of the anti-tumor immune response and production of growth factors. In particular, cancer-associated immune cells such as regulatory T cells (Treg) or MDSCs have been shown to directly inhibit the activity of specific anti-tumor cytotoxic T cell responses (8, 9). In addition, infiltrating inflammatory cells secrete a diverse repertoire of growth factors that can enhance cancer cell proliferation and survival directly [e.g., interleukin (IL)-6 and TNF-α] or by stimulating angiogenesis (10–17). In this context, DCs are very interesting players, especially taking into account their ability to participate in both pro-tumorigenic and anti-tumor processes. For more detailed reviews on DCs in cancer biology and immunotherapy, please refer to Ref. (18–21).

## Immune Properties of Dendritic Cells

Dendritic cells scan peripheral tissues where they recognize, take up, and process antigens and then migrate to lymphoid organs to present antigenic peptides to naive T lymphocytes in the context of major histocompatibility molecules (MHC) (13, 22–24). During this process, DCs become activated, upregulating MHC class II molecules and co-stimulatory molecules such as CD40, CD80, CD86, or OX40L. Upon activation, DCs typically show a decrease in their phagocytic capability, an augment in their efficacy to present processed antigens in the context of MHC molecules, and consequently an improved capability to activate T cells. Through the expression of both MHC class I and II molecules, DCs are able to activate antigen-specific CD8^+^ T cytotoxic and CD4^+^ T helper lymphocytes respectively (25–27). By means of various signals, DCs do not only activate specific T cells, but also drive their differentiation into distinct subsets and even can imprint a migration pattern on these cells toward particular organs or tissues (28). Depending on the stimulus and tissue microenvironment, activated DCs produce an array of cytokines including IL-6, IL-12, IFN-γ, and TNF-α, in addition to several chemokines such as CCL2, CCL3, and CCL5 (29), and thus can play a critical role in shaping the cytokine milieu and leukocyte recruitment and activation.

Dendritic cells are a diverse group of professional antigen-presenting cells that link innate and adaptive immune systems. Several distinct subsets of DCs have been identified and broadly subcategorized into conventional (cDCs) and plasmacytoid (pDCs) (30). Each subset is considered functionally unique, with different TLR expression profiles, response, and outcomes leading to activation of alternate branches of the immune system. For instance, mDC express TLR-2, -4, and -5 whose activation induces IL-12 and IL-6 production. In contrast, pDCs express TLR-7 and -9 ligation resulting in a strong type-I interferon namely IFN-α and are critical players in the innate anti-viral response (31). Such subset differences may have critical implications in success or failure of reprograming cancer-associated DCs *in situ* to generate anti-tumor immune responses.

## Characteristics of Cancer-Associated DCs

The presence of DCs in the stroma of various types of cancer has been well-established (11, 32–35). Interestingly, often these cells do not exert a positive immune influence but act as co-conspirators of tumor growth by inducing regulatory T cell expansion, or directly suppressing T cell responses. These cancer-associated DCs, albeit carrying tumor antigen as we have previously shown (36), express low levels of co-stimulatory molecules (37). Thus, upon encounter with antigen-specific naïve T cells, they can induce an anergic state in these cells favoring tumor immune-escape. This DC phenotype could be caused by products generated by cancer cells or non-cancer cells present in the microenvironment of the tumor. For example, tumor-associated cytokines such as vascular endothelial growth factor (VEGF), IL-10, prostaglandin E-2 (PGE2), and transforming growth factor (TGF)-β can profoundly affect the nature of DCs (38, 39). Indeed, we have previously shown that DCs that were co-opted by the mouse tumors upon injection, acquired angiogenic properties (10). As we have recently reported, the particular characteristics of the extracellular matrix components can also shape the immune properties of these cells (40). Importantly, tumor factors usually exert a systemic effect as previously described (41, 42). For example, it has been demonstrated that VEGF induces a potent systemic effect on both primary and secondary immune organs (41). Therefore, DCs at lymphoid organs can be influenced by tumor factors and/or immunosuppressive leukocytes that can affect their properties (43).

Cancer-associated DCs can also contribute to tumor development by producing factors that promote angiogenesis (44). In the mouse model, we have recently shown that myeloid DCs are able to produce an array of angiogenic molecules *in vitro*, including matrix metalloproteases, VEGF, angiogenin, heparanase, and basic fibroblast growth factors among others (40). We have also previously shown that DC precursors participate in tumor progression and angiogenesis in a mouse model of ovarian cancer (10). Moreover, depletion of cancer-associated DCs *in vivo* was found to reduce tumor growth and decrease angiogenesis in a mouse model of ovarian cancer (45, 46). Not surprisingly, in the same way DCs contributed to angiogenesis in the Lewis lung carcinoma model (47). In humans, cancer-associated DCs have also been shown to produce angiogenic factors and promote neovascularization in the TME (11, 35, 48).

Collectively, these studies provide ample evidence in support of tumors’ capability to reprogram the biology of DCs, inducing them to exert immunosuppressive or angiogenic effects, favoring tumor growth and survival.

## Reprograming Cancer-Associated DC to Induce Anti-Tumor Immunity

The “immune paralysis” of cancer-associated DCs can be overcome in an experimental setting by blocking IL-10R while simultaneously activating specific pattern recognition receptors (PRRs). Upon treatment, the cells regain their ability to activate antigen-specific T cells (10, 49, 50). Considering that cancer-associated DCs can harbor tumor antigen, a compelling strategy would be to reprogram them *in vivo*. Thus, these cells will be transformed into effective antigen-presenting cells capable of promoting anti-tumor immunity and combating tumor growth.

In the mouse model, targeted delivery of antigens to DCs via specific molecules expressed on the DC surface has been investigated. For example, antibodies specific to these surface molecules have been fused with antigens in order to induce an immune response mediated by specific DC populations. Targeting ovalbumin to CD205 and 33D1 molecules on the surface of DCs *in vivo* helped to markedly enhance and qualitatively direct the antigen-presenting properties of CD8+ and CD8− DC subpopulations of splenic DCs. This difference in antigen processing is suggested to be intrinsic to the DC subsets in association with increased expression of proteins involved in MHC processing (51). Likewise, immunization strategies have been designed using antibody–tumor antigen fusion proteins targeting DCs via CD205 (52) or CD11c (53). In addition, antibodies specific to DC surface molecules have been used to coat liposomes or nanoparticles to deliver antigens and inflammatory compounds to DCs *in situ* in a mouse model (54) or to target human DCs (55). Other strategies involve the design of antigen-carrying lentiviral vectors capable of selectively binding to DCs (56).

Evidence that phenotype of cancer-associated DCs can be altered *in vivo* is found in human clinical trials. Anti-tumor therapies using anti-VEFG antibodies, alone or in combination with other drugs, have been evaluated in preclinical and clinical studies (57–60). Interestingly, tumor patients treated with anti-VEGF antibody showed decreased levels of immunosuppressive DCs (61). Similarly, it has been demonstrated that the endothelial cell-produced antiangiogenic cytokine vascular endothelial growth inhibitor induces DC maturation (62). On the other hand, further highlighting the complexity of DC modulation by the TME, cancer patients treated with VEGF-trap [a fusion protein of extracellular domains of VEGF receptor(R)-1 and -2, which can capture all VEGF isoforms] did not show a significant improvement in their immune response, despite a significant increase in the proportion of activated DCs (63). Thus, therapies directly focused on targeting DC *in vivo* must be designed to enhance this effect.

Pioneering research has been performed by the Conejo-Garcia group aimed at reprograming cancer-associated DCs in order to generate a vaccine *in situ* (64). For these studies, a mouse model of ovarian cancer was used. Ovarian cancer characteristically exhibits metastasis within the peritoneal cavity, and is thus an excellent target for localized immunotherapies (65). In a mouse model of ovarian cancer ascites, the group showed that intraperitoneal co-delivery of TLR3 ligands and CD40-activating antibodies induced up-regulation of co-stimulatory molecules in cancer-associated DCs together with increased antigen presentation and anti-tumor T cell response (66). A more focused strategy involved directly targeting cancer-associated DCs with nanoparticles carrying pre-miRNA oligonucleotides that were able to reprogram these immunosuppressive cells into promoters of anti-tumor immune response by increasing miR-155 activity in the targeted cells (67). In addition, similar results were obtained when cancer-associated DCs were targeted by linear polyethylenimine nanoparticles encapsulating non-viral siRNA. These particles were avidly engulfed by the cells, activating them through TLR5 and inducing a potent anti-tumor immune response (64). Lastly, an alternative procedure to activate cancer-associated DCs *in situ* was recently reported. As described by Baird et al. (68), intratumoral administration of an avirulent strain of *Toxoplasma gondii* in a model of ovarian cancer specifically infected cancer-associated DCs (68). These cells reversed their immunosuppressive status and were able to activate a robust anti-tumor T cell response. Finally, future studies will also need to focus on enhancing the migratory capability of reprogramed DCs toward lymph nodes in order to generate a robust T cell response.

## Conclusion

Dendritic cells comprise a population of leukocytes with the capability of activating specific immune responses to promote immunity or induce tolerance. They capture, process, and present antigens thereby activating T cells that carry cognate receptors for these presented antigens. Consequently, DCs serve vital function in initiating adaptive immunity and orchestrating the immune response outcome. The TME can exert undesirable effects on DCs by either rendering them unable to promote specific immune responses, or transforming them into suppressive cells capable of inducing regulatory T cells collectively creating significant obstacles and challenges in cancer immunotherapy. However, ample evidence supports the feasibility to overcome the immune paralysis of cancer-associated DCs. Herein, we summarized our perspective overview of cancer-associated DCs reprograming *in situ* to generate anti-tumor immune responses that will orchestrate a desirable outcome by halting tumor growth and survival. Knowledge of TME, DC biology, and DC response to specific signals will promote the discovery of new strategies for the reprograming of cancer-associated DCs. The fact that cancer-associated DCs harbor tumor antigens also opens up the tantalizing possibility of reprograming these cells *in vivo*, thus inducing a *de facto* patient personalized vaccine. Using innovative approaches to target DCs is vital, and these types of studies will be important in revealing the most effective strategies to overcome setbacks that troubled the field for so long, subsequently helping advance anti-tumor immunotherapy.

## Conflict of Interest Statement

The authors declare that the research was conducted in the absence of any commercial or financial relationships that could be construed as a potential conflict of interest.
